# Insulinoma Mimic: Tramadol-induced Hypoglycemia

**DOI:** 10.1210/jcemcr/luaf034

**Published:** 2025-03-13

**Authors:** Shannon O’Hara, Tyler Hinshaw, Mathew McKenzie, Victoria Belcher, Ian McCoy, Adnan Haider

**Affiliations:** Department of Internal Medicine, West Virginia University School of Medicine, Morgantown, WV 26506, USA; Department of Internal Medicine, West Virginia University School of Medicine, Morgantown, WV 26506, USA; Department of Internal Medicine, West Virginia University School of Medicine, Morgantown, WV 26506, USA; Department of Internal Medicine, West Virginia University School of Medicine, Morgantown, WV 26506, USA; Department of Internal Medicine, West Virginia University School of Medicine, Morgantown, WV 26506, USA; Section of Endocrinology and Metabolism, Department of Internal Medicine, West Virginia University School of Medicine, Morgantown, WV 26505, USA

**Keywords:** hypoglycemia, endogenous hyperinsulinemia, tramadol

## Abstract

Endogenous hyperinsulinemia hypoglycemia has numerous etiologies. The objective of this report is to describe a patient with severe hyperinsulinemic hypoglycemia with no known history of diabetes or hypoglycemia who presented with acute altered mental status. Blood glucose was noted to be 40 mg/dL (2.22 mmol/L) (reference range 65-125 mg/dL; 3.61-6.94 mmol/L). The patient's sulfonylurea screen was negative. Following 1 mg glucagon injection, the patient's glucose did not improve, a response inconsistent with insulinoma. Imaging studies of the pancreas did not show pancreatic mass. Two weeks before the presentation, the patient started on tramadol for back pain with the dose increased 3 days prior to presentation. The patient's hypoglycemia resolved and returned to baseline 4 days after the initial presentation. Tramadol has been reported to cause hypoglycemia, especially in the elderly population. Tramadol may act on μ receptors on β cells to upregulate insulin secretion. When approaching a patient with endogenous hyperinsulinemia, one should consider tramadol or other analgesics as a possible etiology.

## Introduction

Identifying the etiology of hypoglycemia can be challenging, particularly in hospitalized patients with several comorbid conditions. Diagnostic workup is expensive and time-consuming and often requires a complete endocrine evaluation. In critically ill patients, possible etiologies include adrenal insufficiency, alcohol use, starvation, and critical illness. If suspecting endogenous hyperinsulinemia, a supervised fast can be performed to differentiate between these causes by checking insulin, C-peptide, proinsulin, and β-hydroxybutyrate levels during fasting hypoglycemia and screening for oral hypoglycemic agents. A rise in glucose post-glucagon injection before terminating the fast can provide important clues to clinicians and may help direct the resources more efficiently when managing these patients. When hypoglycemia is insulin mediated due to an insulinoma, then glucose levels are expected to rise greater than 25 mg/dL (1.39 mmol/L) within 15 minutes following the glucagon challenge. When the expected glucose response to glucagon is not observed, alternative etiologies like medications should be considered.

## Case Presentation

A 68-year-old male presented to the emergency department from a skilled nursing facility (SNF) for acute encephalopathy over the previous 24 hours. In the emergency room, he was found to be in hypercapnic hypoxic respiratory failure and hypoglycemic with a plasma glucose level of 40 mg/dL (2.22 mmol/L) (reference range 65-125 mg/dL; 3.61-6.94 mmol/L). Medical records from SNF did not show any past medical history of diabetes or hypoglycemia. Additional medical history included chronic obstructive pulmonary disease, obstructive sleep apnea, hypothyroidism, hypertension, atrial fibrillation on apixaban, benign prostatic hypertrophy, anxiety, and depression. The patient had Roux-en-Y gastric bypass (RYGB) 23 years prior for morbid obesity. Information about patient weight at the time of RYGB, nadir weight, and weight regain were not available on account of patient mental status.

Further history was obtained from the nurses taking care of the patient regularly via telephone. The patient reportedly had a good appetite and was getting regular morning metabolic panels, none of which were remarkable for hypoglycemia. About 10 days before admission, he started complaining of back pain and was started on tramadol; the dose escalated to 50 mg every 6 hours a few days before hospitalization.

## Diagnostic Assessment

The patient appeared somnolent and was only able to answer yes/no questions by nodding his head. He had a large body habitus, had a body mass index of 42 kg/m^2^, appeared chronically ill, was pale and in mild respiratory distress, and did not have focal neurologic deficits.

To further assess his hypoglycemia, a supervised fast was completed by holding dextrose-containing fluids and tube feeds. Within 3 hours of stopping fluids and tube feeds, the patient’s capillary glucose was less than 40 mg/dL (2.2 mmol/L), and the serum glucose, C-peptide, serum insulin, proinsulin, sulfonylurea screen, insulin antibodies, and β-hydroxybutyrate were obtained. (Table 1). High C-peptide, insulin, and proinsulin at the time of hypoglycemia were consistent with endogenous hyperinsulinemic hypoglycemia. The patient exhibited no change in plasma glucose 15 minutes after 1 mg glucagon administration—plasma glucose changed from 22 mg/dL (1.22 mmol/L) to 21 mg/dL (1.17 mmol/L) (reference range 65-125 mg/dL; 3.61-6.94 mmol/L) after the administration of glucagon. The lack of increase in plasma glucose following glucagon was not consistent with the expected response seen in conditions associated with high insulin states such as insulinoma. Additional studies included sulfonylurea screen, IGF-1 level, and insulin autoantibody level, all of which were within normal limits.

**Table 1.** Hypoglycemia workup and lab comparison with a known insulinoma case

	Known insulinoma (SI units)	Our patient (SI units)	Reference range (SI units)
Serum glucose	45 mg/dL (2.5 mmol/L)	22 mg/dL (1.22 mmol/L)	65-125 mg/dL (3.61-6.94 mmol/L)
Serum insulin	49.1 mIU/mL (341 pmol/L)	84.9 mIU/mL (589.58 pmol/L)	3.2-32.1 mIU/mL (22.22-222.92 pmol/L)
C-peptide	3.5 ng/mL (1.16 nmol/L)	21.1 ng/mL (7.03 nmol/L)	1.4-6.8 ng/mL (0.47-2.26 nmol/L)
Proinsulin	105.2 mIU/mL (730.61 pmol/L)	30.72 mIU/mL (213.3 pmol/L)	<2.71 mIU/mL (<18.8 pmol/L)
β-hydroxybutyrate	0 mg/dL (0 mmol/L)	0 mg/dL (0 mmol/L)	4.16 mg/dL (<0.4 mmol/L)
IGF-1	189 ng/mL (24.57 nmol/L)	36 ng/mL (4.71 nmol/L)	41-279 ng/mL (5.36-36.48 nmol/L)
Sulfonylurea screen	Negative	Negative	Negative
1 mg glucagon 15 minutes postinjection glucose	45 mg/dL (2.5 mmol/L) rise in glucose	No improvement in plasma glucose	Expected rise of > 25 mg/dL (1.39 mmol/L)
Insulin autoantibody	<0.4 U/mL (<0.4 kU/L)	<0.4 U/mL (<0.4 kU/L)	<0.4 U/mL (<0.4 kU/L)

Abbreviation: SI, International System of Units.

**Figure 1.**
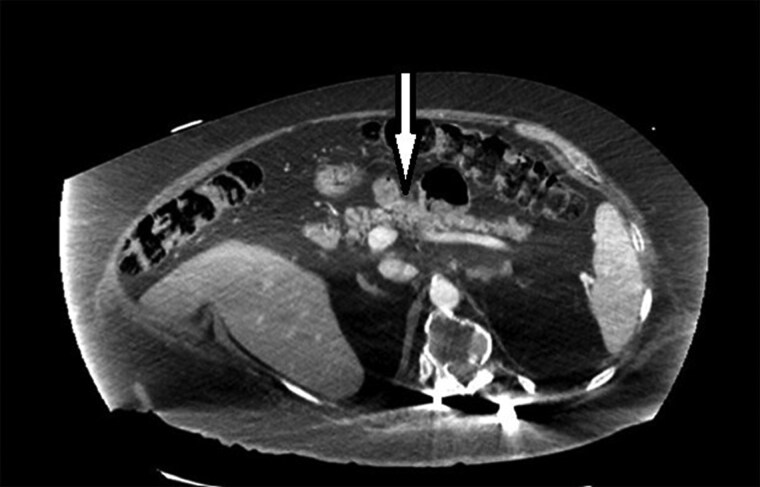
Computed tomography scan of abdomen with/without IV contrast shows fatty infiltration of the pancreas with moderate pancreatic atrophy although no evidence of hyper-enhancing focal masses to suggest insulinoma.

The patient also underwent imaging with computed tomography scan of the abdomen with and without IV contrast, which did not reveal a focal mass consistent with insulinoma (Fig. 1). Magnetic resonance cholangiopancreatography was contraindicated in the patient due to implanted hardware.

## Treatment

The patient was initially managed in the medical intensive care unit due to severe persistent hypoglycemia and encephalopathy. Due to persistent hypoglycemia despite multiple IV glucagon injections and 50 mL 50% dextrose boluses, the patient was started on continuous dextrose-containing fluids. He needed a continuous 20% dextrose solution and therefore required a central line to prevent hypoglycemia in the first 24 hours. The patient was started on Ensure plus tube feeds at 30 cc/hour to reduce the need for dextrose-containing IV fluids.

After completion of hypoglycemia testing and with initial labs pointing to high hyperinsulinemic hypoglycemia, the patient was given 1 dose of subcutaneous octreotide 100 µg with improvement in his serum glucose. Following octreotide, the patient's glucose levels continued to improve without additional doses of octreotide and dextrose-containing IV fluids, and tube feeds were weaned off completely within 24 hours of a single octreotide injection.

Evaluation of his medication list for medications that could potentially be causing hypoglycemia was remarkable only for tramadol. His tramadol was held during his hospital admission in the setting of encephalopathy. After 48 hours of hospitalization, the patient's hypoglycemia resolved with glucose levels ranging between 76 and 281 mg/dL (4.22-15.6 mmol/L) (reference range 65-125 mg/dL; 3.61-6.94 mmol/L), and all dextrose-containing IV fluids and tube feeds were discontinued. Interestingly, the patient also had nothing by mouth for 16 hours during hospitalization 6 days after the initial presentation because of concern for aspiration pneumonia, and he did not develop recurrent hypoglycemia; the patient was not on dextrose-containing IV fluids for this duration.

## Outcome and Follow-up

The workup done during the hypoglycemic spell in our patient was consistent with endogenous hyperinsulinemia. Sulfonylurea ingestion and insulin autoimmune syndrome were also excluded from the blood work. A cortisol level of 13.3 µg/dL (366.9 nmol/L) was reassuring and ruled out adrenal insufficiency. Several clinical findings were not consistent with insulinoma. Our patient did not respond to IV glucagon as plasma glucose concentration did not rise by at least 25 mg/dL (1.4 mmol/L) despite no evidence of hepatic synthetic dysfunction. A rise in glucose of greater than 25 mg/dL (1.4 mmol/L) suggests adequate glycogen stores. Severe malnutrition can also cause glycogen store depletion. However, in our patient this was not the case based on normal albumin and good calorie intake as reported by the nursing home staff taking care of him on a regular basis. Insulin enhances glycogen stores in the liver, and patients with chronic hyperinsulinemia are expected to show a greater than 25 mg/dL rise in blood glucose following glucagon injection. Second, no discrete lesion was identified on the pancreatic computed tomography scan. Although small insulinoma can be missed on imaging studies, in our patient the degree of C-peptide, proinsulin, and insulin elevation were not consistent with small-sized insulinoma. Endoscopic ultrasound was not performed because of the patient history of RYGB surgery, as the endoscopic ultrasound imaging would not be able to provide adequate images of the pancreatic head. Third, just 4 days after the documented hypoglycemic spells (and almost 8 days after the last tramadol dose), the patient did not demonstrate any hypoglycemia without dextrose infusion when he was without anything by mouth for 16 hours because of concerns of aspiration pneumonia. A plasma glucose obtained after 16 hours of fasting showed a glucose level of 92 mg/dL (5.1 mmol/L).

The hypoglycemia workup of a patient who was found to have a 3 cm insulinoma is shown in Table 1, highlighting the difference between inappropriately normal C-peptide, high insulin, and proinsulin in insulinoma patients vs very high values seen in our patient from tramadol.

The patient was discharged back to the same SNF with instructions to avoid tramadol in the future. Continuous glucose monitoring was placed on the patient, and 4 weeks after the episode it did not show any low blood sugar on the monitor.

## Discussion

Tramadol is approved by the US Food and Drug Administration for moderate to severe pain. Tramadol is a commonly prescribed pain medication with 15.5 million prescriptions written in 2021 in the United States. The liver enzyme CYP2D6 converts tramadol to its active metabolite M1 (O-demethyl tramadol), which has a stronger affinity for the μ receptor than the inactive form. The M1 metabolite demonstrates analgesic potency up to 6 times greater than tramadol [1]. The analgesic effect is also attributed to the antinociceptive effect in the descending pathway, secondary to the inhibition of serotonin and norepinephrine reuptake [2].

With the increased use of tramadol, there have been growing incidences of less commonly reported side effects (ie, hypoglycemia). Our case demonstrates evidence of endogenous hyperinsulinemia in a patient who had no localizing pancreatic lesions and reversible hypoglycemia, which started after tramadol was initiated and resolved after stopping tramadol, suggesting tramadol-induced hyperinsulinemic hypoglycemia.

Multiple case reports have demonstrated hypoglycemia either during tramadol overdose [3], within 30 days of starting the medication, or following rapid dose escalation. The frail, elderly population is especially susceptible to the hypoglycemia induced by tramadol [4]. Hyperinsulinemic hypoglycemia is also noted with other pain medications including methadone [5] and dextropropoxyphene [6], especially in patients with advanced chronic kidney disease.

Retrospective analysis from rodent studies shed light on the possible mechanism of tramadol-induced hypoglycemia. In rats with streptozocin-induced diabetes, lowering of plasma glucose was seen in the 30 minutes after IV injection of tramadol. This effect of tramadol was abolished by pretreatment with naloxone [7]. In our case, the hypoglycemia was from endogenous hyperinsulinemia, indicating increased secretion of insulin by the β cells. This finding could be explained by the possibility of μ receptors on the β cells. Opioid peptides and opiate receptors have been demonstrated in pancreatic islet cells [8]. β-endorphin at high dose levels augmented the glucose- and terbutaline-induced insulin secretion in animal models [8]. In another interesting study, streptozotocin-induced diabetic mice were given methadone. Chronic methadone treatment significantly reduced hyperglycemia and the incidence of diabetes and restored pancreatic insulin secretion in these mice, likely indicating the action of μ receptor agonism and increased insulin secretion from the pancreas [9]. In healthy human subjects without diabetes, a 30% reduction in epinephrine response was noted in response to hypoglycemic clamps following morphine infusion as compared to normal saline infusion in the same healthy volunteers, indicating blunting of hypoglycemia response following morphine infusion [10].

In a pharmacovigilance study, tramadol-induced hypoglycemia occurred rapidly after initiation—within 10 days of treatment in 77% of patients [11]. An epidemiological study investigating the association between tramadol use and hospitalization for hypoglycemia found that tramadol use is associated with an increased risk of hospitalization for hypoglycemia, with the highest risk around the time of treatment initiation [12]. A World Health Organization pharmacovigilance database disproportionality analysis performed after excluding concomitant hypoglycemic drugs found that tramadol use was more likely associated with hypoglycemia [13].

The opioid and obesity epidemic has seen the use of an increase in narcotic pain medications. These prescriptions may be written for diabetic patients already on medications that can cause hypoglycemia. Warning the susceptible patients about this potential side effect and increasing blood glucose monitoring will avoid severe hypoglycemia.

Hospitalists, intensivists, and emergency room physicians should be aware of this potential complication that is associated with tramadol use. A detailed history including any new medication use will likely be cost-effective and can avoid prolonged hospitalizations and expensive testing.

## Learning Points

- Increased awareness of tramadol-induced hypoglycemia is important.
- The degree of C-peptide, insulin elevation, in the correct clinical setting of the acute hypoglycemia may serve as a clue to tramadol-induced hypoglycemia.
- Blunted post-glucagon increase in glucose levels is inconsistent with insulinoma and may serve as another clue, indicating that other acute etiologies should be investigated.

## Contributors

All authors made individual contributions to the authorship. S.O. and A.H. were involved in the diagnosis and management of the patient. S.O, T.H, M.M, I.M., and A.H. were involved in manuscript composition. V.B. was involved in the preparation of tables and figures. All authors reviewed and approved the final draft.

## Data Availability

Original data generated and analyzed during this study are included in this published article.

## References

[1] Dayer P, Desmeules J, Collart L. [Pharmacology of tramadol]. Drugs. 1997;53(Supplement 2):18‐24. 9190321 10.2165/00003495-199700532-00006

[2] Albrecht E, Pereira P, Bayon V, et al The relationship between postoperative opioid analgesia and sleep apnea severity in patients undergoing hip arthroplasty: a randomized, controlled, triple-blinded trial. Nat Sci Sleep. 2022;14:303‐310. 35241942 10.2147/NSS.S348834 PMC8887967

[3] Mugunthan N, Davoren P. Danger of hypoglycemia due to acute tramadol poisoning. Endocr Pract. 2012;18(6):e151‐e152. 22982791 10.4158/EP12070.CR

[4] Grandvuillemin A, Jolimoy G, Authier F, Dautriche A, Duhoux F, Sgro C. Hypoglycémie lors d'un traitement par tramadol. A propos de deux cas [tramadol-induced hypoglycemia. 2 cases]. Presse Med. 2006;35(12):1842‐1844. 17159739 10.1016/s0755-4982(06)74913-2

[5] Kanbour S, Balaji A, Chae K, et al Insulinoma mimic: methadone induced hypoglycemia. BMJ Case Rep. 2022;15(7):e245890. 10.1136/bcr-2021-245890 PMC9330285 35882435

[6] Almirall J, Montoliu J, Torras A, Revert L. Propoxyphene-induced hypoglycemia in a patient with chronic renal failure. Nephron. 1989;53(3):273‐275. 2797348 10.1159/000185757

[7] Cheng JT, Liu IM, Chi TC, Tzeng TF, Lu FH, Chang CJ. Plasma glucose–lowering effect of tramadol in streptozotocin-induced diabetic rats. Diabetes. 2001;50(12):2815‐2821. 11723065 10.2337/diabetes.50.12.2815

[8] Ahrén B . Effects of beta-endorphin, met-enkephalin, and dynorphin A on basal and stimulated insulin secretion in the mouse. Int J Pancreatol. 1989;5(2):165‐178. 2574736 10.1007/BF02924417

[9] Amirshahrokhi K, Dehpour AR, Hadjati J, Sotoudeh M, Ghazi-Khansari M. Methadone ameliorates multiple-low-dose streptozotocin-induced type 1 diabetes in mice. Toxicol Appl Pharmacol. 2008;232(1):119‐124. 18671992 10.1016/j.taap.2008.06.020

[10] Carey M, Gospin R, Goyal A, et al Opioid receptor activation impairs hypoglycemic counterregulation in humans. Diabetes. 2017;66(11):2764‐2773. 28860128 10.2337/db16-1478 PMC5652610

[11] Bourne C, Gouraud A, Daveluy A, et al Tramadol and hypoglycaemia: comparison with other step 2 analgesic drugs. Br J Clin Pharmacol. 2013;75(4):1063‐1067. 22943675 10.1111/j.1365-2125.2012.04451.x PMC3612724

[12] Fournier JP, Azoulay L, Yin H, Montastruc JL, Suissa S. Tramadol use and the risk of hospitalization for hypoglycemia in patients with noncancer pain. JAMA Intern Med. 2015;175(2):186‐193. 25485799 10.1001/jamainternmed.2014.6512

[13] De Canecaude C, Rousseau V, Sommet A, Montastruc JL. Tramadol-induced hypoglycemia: a pharmacovigilance study. Fundam Clin Pharmacol. 2021;35(5):933‐936. 33511683 10.1111/fcp.12655

